# SHIP represses lung inflammation and inhibits mammary tumor metastasis in BALB/c mice

**DOI:** 10.18632/oncotarget.6611

**Published:** 2015-12-14

**Authors:** Melisa J. Hamilton, Elizabeth C. Halvorsen, Nancy E. LePard, Momir Bosiljcic, Victor W. Ho, Vivian Lam, Judit Banáth, Kevin L. Bennewith, Gerald Krystal

**Affiliations:** ^1^ Department of Integrative Oncology, British Columbia Cancer Agency Research Centre, Vancouver, BC, Canada; ^2^ Terry Fox Laboratory, British Columbia Cancer Agency Research Centre, Vancouver, BC, Canada

**Keywords:** SHIP, BALB/c, 4T1, metastasis, myeloid cells

## Abstract

SH2-containing-inositol-5′-phosphatase (SHIP) is a negative regulator of the phosphatidylinositol-3-kinase pathway in hematopoietic cells and limits the development of leukemias and lymphomas. The potential role of SHIP in solid tumor development and metastasis remains unknown. While SHIP restricts the aberrant development of myeloid cells in C57BL/6 mice, there are conflicting reports regarding the effect of SHIP deletion in BALB/c mice with important consequences for determining the influence of SHIP in different model tumor systems. We generated SHIP−/− BALB/c mice and challenged them with syngeneic non-metastatic 67NR or metastatic 4T1 mammary tumors. We demonstrate that SHIP restricts the development, alternative-activation, and immunosuppressive function of myeloid cells in tumor-free and tumor-bearing BALB/c mice. Tumor-free SHIP−/− BALB/c mice exhibited pulmonary inflammation, myeloid hyperplasia, and M2-polarized macrophages and this phenotype was greatly exacerbated by 4T1, but not 67NR, tumors. 4T1-bearing SHIP−/− mice rapidly lost weight and died from necrohemorrhagic inflammatory pulmonary disease, characterized by massive infiltration of pulmonary macrophages and myeloid-derived suppressor cells that were more M2-polarized and immunosuppressive than wild-type cells. Importantly, while SHIP loss did not affect primary tumor growth, 4T1-bearing SHIP−/− mice had 7.5-fold more metastatic tumor cells in their lungs than wild-type mice, consistent with the influence of immunosuppressive myeloid cells on metastatic growth. Our findings identify the hematopoietic cell-restricted protein SHIP as an intriguing target to influence the development of solid tumor metastases, and support development of SHIP agonists to prevent the accumulation of immunosuppressive myeloid cells and tumor metastases in the lungs to improve treatment of metastatic breast cancer.

## INTRODUCTION

The src homology 2-containing inositol-5′-phosphatase, SHIP (also called SHIP1), is a negative regulator of proliferation, differentiation, survival, motility, and activation that is expressed primarily in hematopoietic cells [1, 2]. SHIP is thought to carry out its biological effects by hydrolyzing the critical phosphatidylinositol-3-kinase (PI3K)-generated second messenger, phosphatidylinositol-3,4,5-trisphosphate (PIP_3_) [1, 2]. However, unlike the more ubiquitous tumor suppressor phosphatase and tensin homolog (PTEN), which hydrolyses PIP_3_ to PI-4,5-P_2_, SHIP hydrolyzes PIP_3_ to PI-3,4-P_2_ and thus likely has biological effects distinct from PTEN [3, 4].

SHIP−/− C57BL/6 mice are viable and fertile, but have a shortened lifespan, hunched posture, and thinner bodies than wild-type (WT) littermates [5]. This failure of SHIP−/− mice to thrive is likely due, at least in part, to massive myeloid cell infiltration of the lungs [5, 6]. SHIP−/− C57BL/6 mice also have elevated numbers of hyper-responsive pulmonary mast cells, which play a significant role in the elevated lung tissue levels of histamine and T_H_2 cytokines and chemokines, as well as the chronic lung inflammation that characterizes these mice [6, 7]. In addition, SHIP−/− C57BL/6 mice possess an increased number of hyper-resorptive osteoclasts and, consequently, suffer from severe osteoporosis [8]. A critical feature of SHIP−/− C57BL/6 mice is that their myeloid progenitors are significantly more responsive to low levels of cytokines, growth factors, and chemokines than their WT counterparts [5, 9]. This explains why SHIP−/− mice overproduce granulocytes, mast cells, and macrophages (Mϕs), and also force erythropoiesis out of the bone marrow and into the spleen and elsewhere [5, 7]. As a result, SHIP−/− mice develop splenomegaly, due to both extramedullary erythropoiesis and myelopoiesis [5].

Since the majority of studies examining the role of SHIP in hematopoiesis have been performed using SHIP−/− mice on a C57BL/6 genetic background, the role of SHIP in other genetic backgrounds remains unclear. In 2010, Roongapinun *et al.* reported that SHIP−/− BALB/c mice exhibit far less, albeit detectable, lung inflammation compared to SHIP−/− C57BL/6 mice [10]. This was unexpected since BALB/c mice are more M2 and T_H_2 prone than C57BL/6 mice [11] and asthmatic lung inflammation is considered a T_H_2 condition [12]. In 2011, Maxwell *et al.* also reported that deleting SHIP in BALB/c mice results in a markedly reduced pathology compared to SHIP−/− C57BL/6 mice, however, they found no evidence of any inflammatory lung disease or increased myelopoiesis in these mice [13]. Thus, the effect of SHIP deletion in BALB/c mice is somewhat unclear, and has important consequences for determining the role of SHIP in tumor development and growth in different model systems.

SHIP acts as a tumor suppressor in hematopoietic malignancies by directly restraining the PI3K pathway within SHIP-expressing leukemia and lymphoma cells. Hyperactivity of the PI3K pathway is a characteristic of many cancers [14] and inactivating mutations of SHIP or a reduction in SHIP levels have been associated with both human and murine leukemias and lymphomas, including acute lymphoblastic leukemia [15], diffuse large B cell lymphoma [16, 17], acute myeloid leukemia [18] and erythroleukemia [19]. The role of SHIP in solid tumor development has been less well-studied, although SHIP is known to influence the development and function of immune cell populations that can affect solid tumor growth. SHIP limits the response of immune cells to cytokines, chemokines, and growth factors, and specifically restricts the expansion and activity of myeloid-derived suppressor cells (MDSCs) [20, 21], M2 Mϕs [22], and regulatory T cells (Tregs) [23]. Each of these cell types exhibits pro-tumorigenic functions in model tumor systems, including the suppression of anti-tumor T cell-mediated immune responses [24, 25]. Consistent with the role of SHIP in restricting myeloid cell development and the influence of myeloid cells on solid tumor growth, the reduced expression or absence of SHIP in myeloid cells has been associated with increased growth of Panc02 tumors [21] and Lewis lung carcinoma (LLC) tumors [22] in C57BL/6 mice, respectively. However, the effect of SHIP loss on tumor growth in non-C57BL/6 genetic backgrounds and the potential role of SHIP in solid tumor metastasis are unknown.

Metastatic mammary tumors can induce an M2 phenotype in myeloid cells through the production of G-CSF [26, 27] and other cytokines [28]. We, and others, have established that immunosuppressive MDSCs and M2 Mϕs promote the development and spread of mammary tumors [25, 29–32]. We have also shown that Mϕs can be 30-fold more potent suppressors of activated T cell proliferation than MDSCs, and that elevated levels of Mϕs in the lungs promote metastatic tumor growth [29]. Since SHIP is known to restrict the development of a tumor-promoting phenotype in myeloid cells in C57BL/6 mice, we wanted to determine whether the absence of SHIP would alter the growth and/or metastasis of murine mammary tumors. We were also curious whether the presence of mammary tumors would induce phenotypic changes in SHIP−/− BALB/c mice.

We report herein that tumor-free SHIP−/− BALB/c mice exhibit pulmonary inflammation and myeloid hyperplasia that is greatly exacerbated upon challenge with orthotopic 4T1 metastatic mammary tumors. Moreover, 4T1 mammary tumors, but not 67NR non-metastatic mammary tumors, cause SHIP−/− BALB/c mice to die of necrohemorrhagic inflammatory pulmonary disease within 17 days of tumor implantation. These 4T1 tumor-bearing SHIP−/− mice possess higher levels of myeloid cells that are more M2-skewed and more immunosuppressive than myeloid cells from WT mice bearing 4T1 tumors. Importantly, we found that mammary tumor metastases are also dramatically increased in the lungs of SHIP−/− mice. These data indicate that SHIP restricts mammary tumor metastases in BALB/c mice and support the development of SHIP agonists as a viable therapeutic strategy to decrease pulmonary metastases in breast cancer.

## RESULTS

### SHIP−/− BALB/c mice display pulmonary inflammation and elevated levels of M2-polarized, immunosuppressive myeloid cells

**Figure 1:**
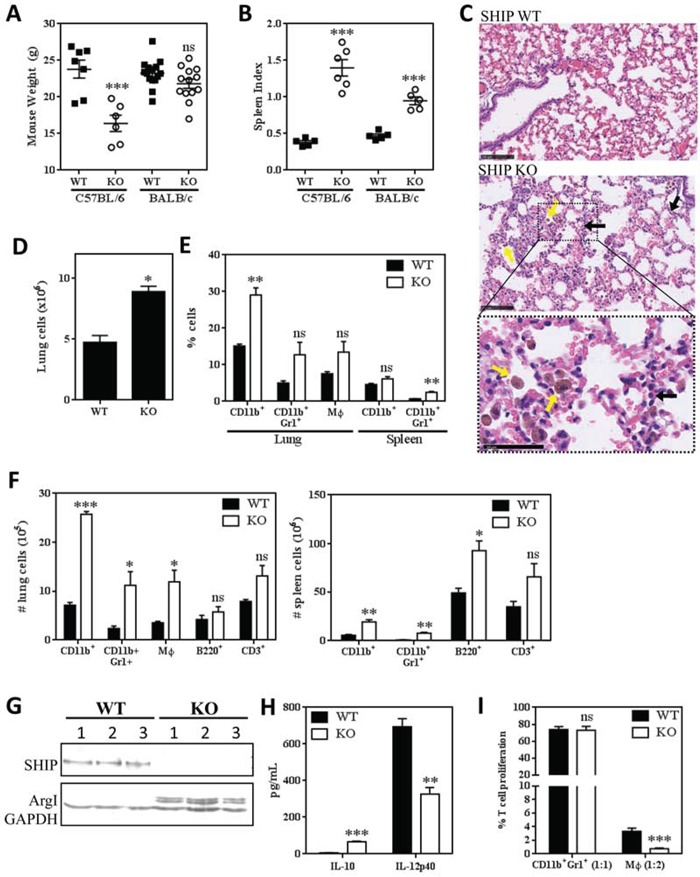
SHIP represses pulmonary inflammation and Mϕ M2 polarization and immunosuppression in BALB/c mice **A.** Body weights of WT and SHIP−/− C57BL/6 and BALB/c mice. **B.** Spleen indices (spleen weight as a proportion of body weight) of WT and SHIP−/− C57BL/6 and BALB/c mice. Each symbol in A and B represents an individual mouse. **C.** Lung sections of WT and SHIP−/− BALB/c mice stained with H&E. SHIP−/− lungs contain increased numbers of Mϕs (yellow arrows) and granulocytes (black arrows). Bottom panel is a magnified region of the SHIP−/− lung section to more clearly show cell morphology. Images are representative of 2 mice from each group; scale bars are 100μm in the first two panels and 50μm in the bottom panel. **D.** Total cells isolated from disaggregated lungs of WT and SHIP−/− BALB/c mice. **E.** Proportions of total CD11b+ cells, CD11b+Gr1+ myeloid cells, and CD11b+F4/80+ Mϕs in the lungs or spleens of WT and SHIP−/− mice. **F.** Numbers of CD11b+ cells, CD11b+Gr1+ myeloid cells, CD11b+F4/80+ Mϕs, B220+ B lymphocytes, and CD3+ T lymphocytes in the lungs (left) or spleens (right) of WT and SHIP−/− mice. Data in D-F are the mean ± SEM with 3-4 mice per group. **G.** Peritoneal Mϕs were harvested from WT or SHIP−/− BALB/c mice and subjected to Western blot analysis. Each lane represents an individual mouse; GAPDH included as a loading control. **H.** WT and SHIP−/− peritoneal Mϕs were stimulated with 100 ng/mL LPS for 3 h and IL-10 and IL-12p40 quantified by ELISA. **I.** CD11b+Gr1+ myeloid cells and CD11b+F4/80+ Mϕs were isolated from the lungs of WT or SHIP−/− mice and co-cultured with anti-CD3 + anti-CD28 stimulated splenocytes (1 CD11b+Gr1+ cell:1 splenocyte; 1 Mϕ:2 splenocytes). Data are expressed as the percent of stimulated T cell proliferation in the absence of myeloid cells (i.e., responders alone) and are the mean ± SEM of two independent experiments, each performed in triplicate. *p <0.05; **p <0.01; ***p <0.001; ns, no significant difference. Significance compared to WT.

Most murine studies elucidating the functions of SHIP have been performed using C57BL/6 mice and the roles of SHIP in other genetic backgrounds remain unclear. To determine the effect of SHIP deletion in BALB/c mice, we backcrossed F2 SHIP−/− mice on a mixed C57BL/6 × 129Sv background for 10 generations onto either C57BL/6 or BALB/c genetic backgrounds and compared their phenotypes. Consistent with Maxwell *et al.* [13], while SHIP−/− C57BL/6 mice were lighter and more frail than WT mice, there was no difference in body weight or general appearance between WT and SHIP−/− BALB/c mice (Fig. 1A). However, we found that SHIP−/− BALB/c mice exhibited splenomegaly compared to their WT counterparts, albeit not as severely as in SHIP−/− C57BL/6 mice (Fig. 1B). We performed histological analyses of lungs from WT and SHIP−/− BALB/c mice and found that SHIP−/− mice exhibited mild lung pathology, characterized by diffuse myeloid inflammation, abnormal pulmonary architecture, and mild hemorrhaging (Fig. 1C). Specifically, the lungs of SHIP−/− BALB/c mice contained increased numbers of Mϕs and neutrophils, poorly inflated alveoli, and hypercellular, thickened alveolar walls (Fig. 1C). Flow cytometric analyses of resected lung tissue confirmed the histological data, in that we observed significant pulmonary hypercellularity (Fig. 1D) along with increased proportions of CD11b^+^ myeloid cells in SHIP−/− BALB/c mice (Fig. 1E). We found increased numbers of pulmonary (Fig. 1F; *left panel*) and/or splenic (*right panel*) CD11b^+^ cells, including both CD11b^+^Gr1^+^F4/80^−^ myeloid cells and CD11b^+^Gr1^−^F4/80^+^ Mϕs, and increased B220^+^ B cells in the spleen. Bone marrow from SHIP−/− BALB/c mice also contained a higher ratio of myeloid to erythroid cells than in WT mice (data not shown), consistent with a myeloproliferative phenotype. Collectively, these data contrast with Maxwell *et al.* [13], and suggest that SHIP−/− BALB/c mice exhibit lung inflammation and myeloid hyperplasia.

To further investigate the myeloid phenotype of SHIP−/− BALB/c mice, we isolated peritoneal Mϕs from WT and SHIP−/− mice. We found that only SHIP−/− Mϕs expressed detectable levels of arginase (Arg) 1 (Fig. 1G) and that SHIP−/− Mϕs produced higher levels of IL-10 and lower levels of IL-12 than WT Mϕs upon LPS stimulation (Fig. 1H), suggesting that SHIP deficiency promotes an M2-skewed phenotype in these mice. Given that Arg1 expression is one mechanism by which myeloid cells can suppress T cell responses [25], we assayed the immunosuppressive properties of both Mϕs and CD11b^+^Gr1^+^ cells isolated from the lungs of WT and SHIP−/− BALB/c mice. Interestingly, CD11b^+^Gr1^+^ cells from naïve WT or SHIP−/− mice did not exhibit significant immunosuppressive effects (Fig. 1I), consistent with our previous findings that CD11b^+^Gr1^+^ cells must first be activated (i.e., by pro-inflammatory or tumor-derived factors) before acquiring suppressive abilities that are indicative of CD11b^+^Gr1^+^ MDSCs [29]. In contrast, both WT and SHIP−/− pulmonary Mϕs exhibited potent immune suppressive properties, with SHIP−/− Mϕs being more suppressive on a per cell basis (Fig. 1I). These findings were consistent with peritoneal Mϕs isolated from C57BL/6 mice, with SHIP−/− Mϕs being more potent suppressors than WT Mϕs in both mouse strains (Supplemental Fig. 1). Collectively, these results indicate that SHIP restricts the numbers of myeloid cells, and the M2 polarization and immune suppressive function of macrophages, in BALB/c mice.

### Non-metastatic 67NR mammary tumors do not dramatically alter the phenotype of SHIP−/− mice

**Figure 2:**
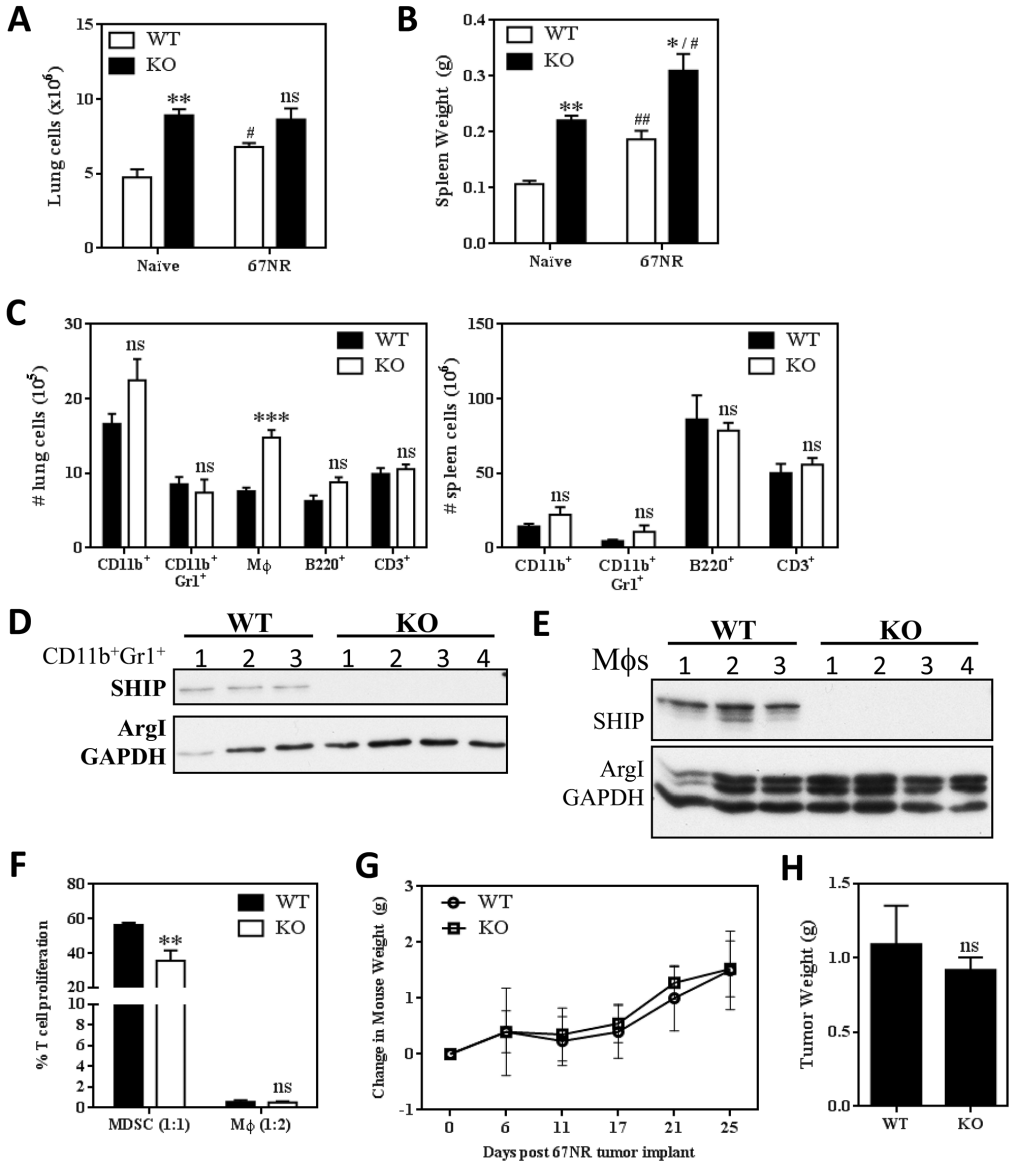
SHIP deficiency does not accelerate the growth of non-metastatic 67NR mammary tumors **A.** Lung cellularity and **B.** spleen weights of naïve (tumor-free) or 67NR tumor-bearing WT or SHIP−/− mice. **C.** Numbers of CD11b+ cells, CD11b+Gr1+ myeloid cells, CD11b+F4/80+ Mϕs, B220+ B lymphocytes, and CD3+ T lymphocytes in the lungs (left) or spleens (right) of WT and SHIP −/− 67NR tumor-bearing mice. Data in A-C are mean ± SEM with 3-4 mice per group. **D.** Pulmonary CD11b+Gr1+ cells and **E.** peritoneal Mϕs were isolated from 67NR tumor-bearing WT and SHIP−/− mice and subjected to Western blot analysis. D-E, each lane represents an individual mouse; GAPDH included as a loading control. **F.** CD11b+Gr1+ cells and CD11b+Gr1-F4/80+ Mϕs were isolated from the lungs of 67NR tumor-bearing WT or SHIP−/− mice and co-cultured with anti-CD3 + anti-CD28 stimulated splenocytes (1 CD11b+Gr1+ cell:1 splenocyte; 1 Mϕ:2 splenocytes). Data are expressed as the fraction of stimulated T cell proliferation in the absence of myeloid cells and are the mean ± SEM of triplicate wells with 3-4 mice per group. **G.** Weight of WT and SHIP−/− mice following 67NR tumor implantation. **H.** 67NR tumors were excised from WT or SHIP−/− mice 21 days after implantation and weighed. G-H, Data are expressed as the mean ± SEM with 3-4 mice per group. */#p <0.05; **/##p <0.01; ***p <0.001; ns, no significant difference. *'s, significance compared to WT mice of the same tumor status; #'s, significance compared to naïve (tumor-free) mice of the same genotype.

Although the loss of SHIP in BALB/c mice causes myeloid hyperplasia and mild pulmonary disease, it does not impair the overall health or lifespan of the animals, at least within the specific pathogen-free environment of our animal facility. To investigate the effect of a challenge on the SHIP−/− BALB/c phenotype and to investigate the role of SHIP on primary and metastatic tumor growth, we orthotopically injected BALB/c mice with syngeneic, non-metastatic 67NR or metastatic 4T1 murine mammary tumors. As shown in Fig. 2A, orthotopic 67NR tumors increased lung cellularity in WT mice and induced modest splenomegaly in both WT and SHIP−/− BALB/c mice (Fig. 2B), although the relative differences were comparable between 67NR-bearing and naïve (tumor-free) WT and SHIP−/− mice. SHIP−/− mice with 67NR tumors had increased numbers (Fig. 2C) and proportions (Supplemental Fig. 2A) of pulmonary Mϕs and decreased proportions of splenic lymphocytes (Supplemental Fig. 2A), although the absolute numbers of B cells and T cells in the spleen were not reduced (Fig. 2C) due to the hypercellular splenomegaly in these mice (Fig. 2B).

To determine whether SHIP regulated M1/M2 polarization in the 67NR tumor model, we isolated pulmonary CD11b^+^Gr1^+^ cells and peritoneal Mϕs to assess Arg1 expression. While CD11b^+^Gr1^+^ cells from the lungs of 67NR-bearing WT or SHIP−/− mice did not display detectable levels of Arg1 (Fig. 2D), the peritoneal Mϕs from both genotypes expressed Arg1 (Fig. 2E). When taken with the data in Fig. 1G indicating Arg1 is not expressed in peritoneal Mϕs from naïve WT mice, these data suggest that 67NR tumors induce Arg1 expression in WT Mϕs.

We then tested the immunosuppressive properties of myeloid cell subpopulations isolated from the lungs of WT or SHIP−/− mice bearing 67NR tumors. We found that CD11b^+^Gr1^+^ cells from 67NR-bearing SHIP−/− mice modestly suppressed T cell proliferation and therefore could be termed MDSCs, and that SHIP−/− MDSCs were more suppressive than WT CD11b+Gr1+ cells on a per cell basis (Fig. 2F). Pulmonary CD11b^+^Gr1^−^F4/80^+^ Mϕs from 67NR-bearing WT and SHIP−/− mice potently inhibited T cell proliferation (Fig. 2F), consistent with our previous findings that Mϕs are more potent immune suppressors than MDSCs in multiple tumor models [29].

We previously found that SHIP−/− C57BL/6 mice with subcutaneous LLC tumors exhibited increased weight loss, morbidity, and faster primary tumor growth than their WT counterparts [22]. However, SHIP−/− BALB/c mice bearing non-metastatic 67NR mammary tumors continued to gain weight at the same rate as WT mice (Fig. 2G) and there were no significant differences in morbidity (data not shown) or primary tumor growth (Fig. 2H). These results suggest that SHIP deficiency on a BALB/c background does not exacerbate the growth of 67NR primary tumors, and that 67NR tumors do not markedly alter the gross phenotype of SHIP−/− BALB/c mice.

### Metastatic 4T1 mammary tumors induce a massive expansion of myeloid cells in the spleens and lungs of SHIP−/− mice

**Figure 3:**
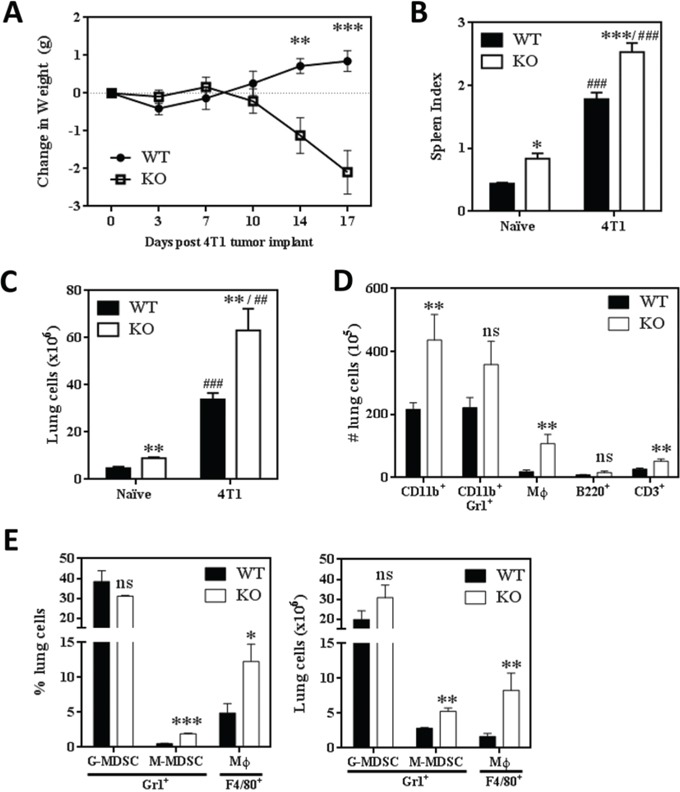
SHIP−/− BALB/c mice challenged with 4T1 metastatic mammary tumors exhibit extreme myeloid hyperplasia **A.** Change in WT or SHIP−/− mouse weights following 4T1 tumor implantation. Data are expressed as the mean ± SEM with a minimum of 10 mice per time point. **B.** Spleen indices (spleen weight / mouse weight) of 4T1 tumor-bearing WT and SHIP−/− mice 15 days after tumor implantation. **C.** Total lung cells isolated from naïve (tumor-free) and 4T1 tumor-bearing WT or SHIP−/− BALB/c mice. **D.** Numbers of CD11b+ cells, CD11b+Gr1+ myeloid cells, CD11b+F4/80+ Mϕs, B220+ B lymphocytes, and CD3+ T lymphocytes in the lungs of WT and SHIP−/− 4T1 tumor-bearing mice. B-D, Data are expressed as the mean ± SEM of two independent experiments with 8-10 mice per group. **E.** Proportion (left) and number (right) of CD11b+Gr1+Ly6G+Ly6Cmid G-MDSCs, CD11b+Gr1+Ly6G-Ly6C+ M-MDSCs, and CD11b+Gr1-F4/80+ Mϕs in the lungs of 4T1 tumor-bearing WT or SHIP−/− mice. *p <0.05; **/##p <0.01; ***/###p <0.001; ns, no significant difference. *'s, significance compared to WT mice of the same tumor status; #'s, significance compared to naïve (tumor-free) mice of the same genotype.

We obtained markedly different results when WT and SHIP−/− BALB/c mice were orthotopically implanted with metastatic 4T1 mammary tumors. Strikingly, while WT mice continued to steadily gain weight, SHIP−/− mice rapidly lost weight beginning 10 days after primary tumor implantation (Fig. 3A). Consistent with previous reports, 4T1 tumors induced splenomegaly in WT mice [29, 33], and we found the splenomegaly was exacerbated in SHIP−/− mice (Fig. 3B). Although there was no difference in absolute spleen weight measured 15 days after tumor implantation (Supplemental Figure 2B), SHIP−/− mice had a much higher splenic index (ratio of spleen weight to body weight) compared to WT mice (Fig. 3B), due to the extreme weight loss observed in the SHIP−/− BALB/c mice. In addition, both 4T1 tumor-bearing WT and SHIP−/− mice had 7-fold more lung cells than naïve WT and SHIP−/− mice (Fig. 3C). This increase in total cell number was reflected in higher numbers of pulmonary CD11b^+^ cells and Mϕs in 4T1-bearing SHIP−/− mice (Fig. 3D), but there was little difference in the relative proportions of myeloid and lymphoid cells in the lungs or spleen (Supplemental Fig. 2C,D). Comparison of cell surface marker expression on myeloid cells isolated from the lungs of WT and SHIP−/− 4T1 tumor-bearing mice revealed that WT CD11b^+^Gr1^+^ cells expressed higher levels of Gr1 than SHIP−/− CD11b^+^Gr1^+^ cells (Supplemental Fig. 3A), while SHIP−/− Mϕs exhibited higher expression of F4/80 (Supplemental Fig. 3B).

The majority of CD11b^+^Gr1^+^ MDSCs induced by the 4T1 tumor model are granulocytic-MDSCs (G-MDSCs) rather than monocytic-MDSCs (M-MDSCs) [29]. To determine whether SHIP deficiency preferentially altered the induction of G-MDSCs or M-MDSCs, we examined the different myeloid subpopulations in 4T1 tumor-bearing WT and SHIP−/− mice. We found that while the majority of CD11b^+^ cells were Ly6G^+^Ly6C^mid^ G-MDSCs in both WT and SHIP−/− mice, pulmonary Ly6G^−^Ly6C^+^ M-MDSCs were significantly increased in SHIP−/− mice (Fig. 3E). SHIP−/− mice also had higher proportions and numbers of Mϕs in the lungs (Fig. 3E). These data were consistent with morphological analyses of disaggregated lung cells, which revealed three distinct subpopulations of pulmonary myeloid cells (Supplemental Fig. 3C). Consistent with the flow cytometry data, the majority of myeloid cells exhibited a granulocytic phenotype (G-MDSCs). Similarly, cells resembling monocytes (likely M-MDSCs) and Mϕs were isolated from the lungs of both WT and SHIP−/− 4T1-bearing mice, although there were many more Mϕs in the lungs of SHIP−/− mice (Supplemental Fig. 3C). Collectively, these data indicate that the loss of SHIP enhances the 4T1-mediated induction of pulmonary myeloid cells, particularly M-MDSCs and Mϕs.

### SHIP−/− myeloid cells induced by 4T1 tumors are alternatively activated and highly immunosuppressive

**Figure 4:**
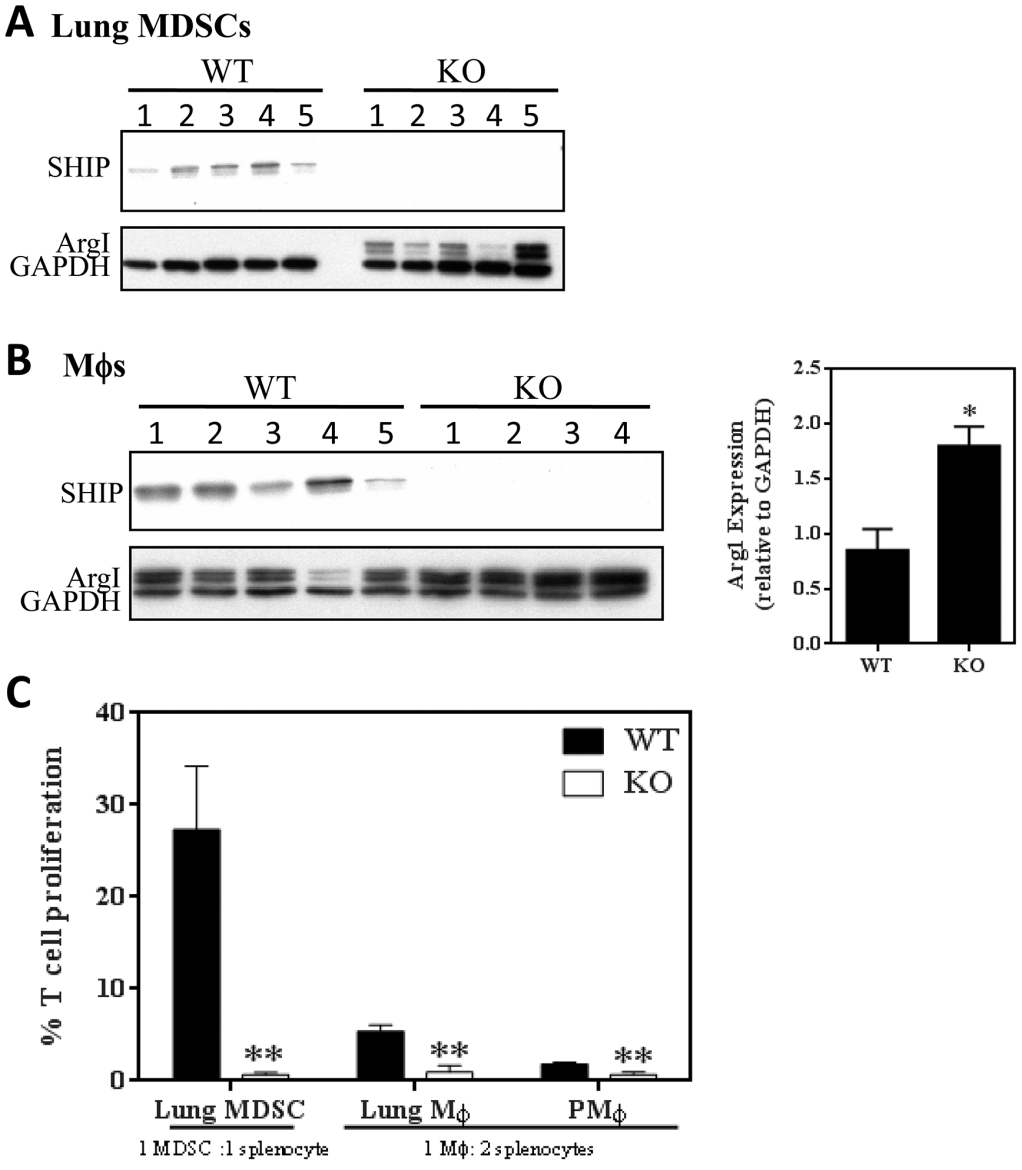
SHIP restricts the immunosuppressive properties of 4T1-induced myeloid cells **A.** CD11b+Gr1+ pulmonary MDSCs or **B.** peritoneal Mϕs were isolated from WT and SHIP−/− 4T1 tumor-bearing mice and subjected to Western blot analysis. For peritoneal Mϕs, Arg1 expression by Western blot (left) was quantified by densitometry and normalized to GAPDH expression (right). A-B, each lane represents an individual mouse. **C.** Pulmonary CD11b+Gr1+ cells, CD11b+Gr1-F4/80+ pulmonary Mϕs, and CD11b+Gr1-F4/80+ peritoneal Mϕs were isolated from WT and SHIP−/− 4T1 tumor-bearing mice and co-cultured with polyclonal-stimulated splenocytes (1 CD11b+Gr1+cell:1 splenocyte; 1 Mϕ:2 splenocytes). Data are expressed as the fraction of stimulated T cell proliferation in the absence of myeloid cells. *p <0.05; **p <0.01. Significance compared to WT.

To determine the functional relevance of myeloid cells in the lungs of 4T1 tumor-bearing mice, we isolated MDSCs and Mϕs from WT and SHIP−/− mice bearing 4T1 tumors. Interestingly, pulmonary MDSCs from 4T1 tumor-bearing SHIP−/− mice, but not WT mice, displayed Arg1 expression (Fig. 4A). We also found that while 4T1 tumors induced Arg1 expression in Mϕs from both WT and SHIP−/− mice (Fig. 4B, *left panel*), the SHIP−/− Mϕs expressed 1.7-fold higher Arg1 levels than WT Mϕs (Fig. 4B, *right panel*), suggesting that SHIP restricts 4T1-induced Arg1 expression in CD11b^+^Gr1^+^ cells and Mϕs. Importantly, we found that SHIP deficiency greatly enhanced the immune suppressive properties of myeloid cells isolated from the lungs of 4T1 tumor-bearing mice (Fig. 4C). While pulmonary CD11b+Gr1+ cells from WT and SHIP−/− mice bearing 4T1 tumors both demonstrated suppressive functions, and therefore are MDSCs SHIP−/− MDSCs were more than 40 times more suppressive on a per cell basis (Fig. 4C). Similarly, both lung and peritoneal Mϕs from 4T1-bearing SHIP−/− mice were more potent suppressors than their WT counterparts (Fig. 4C). Taken together, these data indicate that both 4T1 tumors and SHIP deficiency augment the immunosuppressive potency of pulmonary myeloid cells.

### SHIP−/− mice with 4T1 tumors exhibit necrohemorrhagic inflammatory pulmonary disease and increased pulmonary metastases

**Figure 5:**
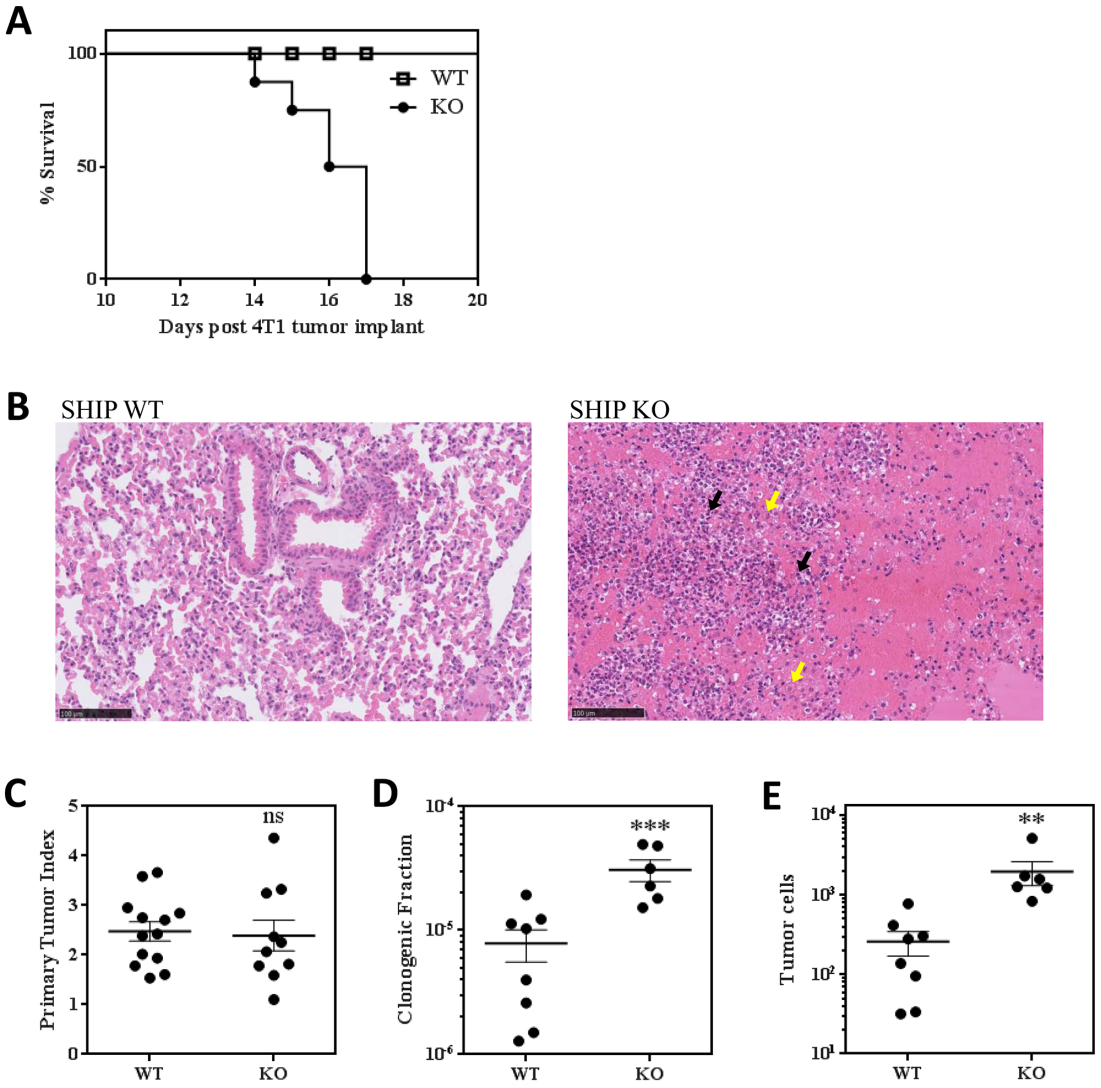
SHIP−/− BALB/c mice challenged with 4T1 tumors exhibit increased lung metastases and rapid death **A.** Survival curve of 4T1 tumor-bearing WT and SHIP−/− mice. Data are the mean ± SEM of 3 independent experiments with a total of 16 mice per group. **B.** Lung sections of 4T1 tumor-bearing WT and SHIP−/− mice stained with H&E. Inflammatory granulocytes (black arrows; likely G-MDSCs) and Mϕs (yellow arrows) are indicated. Images are representative of 2 mice from each group 15 days after 4T1 tumor implantation. **C.** Primary tumor indices (tumor weight / body weight) for WT and SHIP−/− mice 15 days after orthotopic 4T1 tumor implantation. **D.** Proportion of lung cells that give rise to tumor colonies in a colony-forming assay, and **E.** total number of metastatic tumor cells in the lungs of WT and SHIP−/− mice 15 days after 4T1 tumor implantation. C-E, each point represents an individual mouse; bars are mean ± SEM of two independent experiments. **p <0.01; ***p <0.001; ns, no significant difference. Significance compared to WT.

While we did not observe any gross phenotypic differences between WT or SHIP−/− BALB/c mice without tumors or with 67NR tumors, we found that, in addition to dramatic weight loss (Fig. 3A), 4T1 tumors induced the development of red and inflamed ears, hind paws, and tails in SHIP−/− mice (data not shown). Notably, while 100% of WT mice implanted with 4T1 tumors survive for 28 days (at which time they are euthanized due to primary tumor burden), SHIP−/− mice began to die at 14 days after tumor implantation and none of the mice survived past day 17 (Fig. 5A). We investigated the cause of this rapid mortality and found that 4T1 tumor-bearing SHIP−/− mice had severe necrohemorrhagic inflammatory pulmonary disease, which suggested respiratory failure as the likely cause of death. While the lungs of 4T1 tumor-bearing WT mice displayed normal alveolar architecture and no evidence of inflammation, the lungs of SHIP−/− mice were consolidated and exhibited complete loss of alveolar architecture due to severe necrosis, hemorrhage, and inflammatory infiltrates of large numbers of granulocytes and Mϕs (Fig. 5B).

Histological analysis of spleens confirmed that splenic enlargement in both WT and SHIP−/− mice was due to hyperplasia of hematopoietic cells, likely in response to 4T1 tumor-derived factors. While WT spleens showed moderate myeloid, erythroid and megakaryocytic hyperplasia and normal organization 15 days after 4T1 tumor implantation (Supplemental Fig. 4A), the hematopoiesis observed in SHIP−/− spleens was overwhelmingly myeloid in nature and was accompanied by a loss of typical architecture (Supplemental Fig. 4B), suggesting deregulation of myelopoiesis in the absence of SHIP.

We also investigated the effect of SHIP deficiency on 4T1 primary tumor growth and found that there was no difference in the ratio of tumor weight to mouse weight between WT and SHIP−/− mice (Fig. 5C). However, we observed a striking increase in lung metastasis in SHIP−/− mice. Consistent with the known role of myeloid cells in promoting 4T1 tumor metastasis [26, 27, 29], we observed isolated regions of metastatic mammary carcinoma cells in the lungs of SHIP−/− mice (Supplemental Fig. 4C), while there were no obvious microscopic pulmonary metastases in WT mice 15 days after 4T1 tumor implantation. We also performed sensitive clonogenic assays on cells isolated from the lungs of tumor-bearing mice and found that the lungs of SHIP−/− mice possessed a higher proportion of clonogenic cells (Fig. 5D) that translated into 7.5-fold more metastatic tumor cells (Fig. 5E) than the lungs of WT mice 15 days after primary tumor implantation. Taken together, these data demonstrate that SHIP restricts the development and immunosuppressive properties of myeloid cells, protects against severe necrohemorrhagic inflammatory pulmonary disease in BALB/c mice with 4T1 tumors, and represses the development of mammary tumor metastases.

## DISCUSSION

C57BL/6 and BALB/c mice are T_H_1- and T_H_2-skewed mouse models, respectively [34], due to differences in their genetic backgrounds. While C57BL/6 mice are typically more susceptible to T_H_1-driven autoimmune diseases, T_H_2-dominant BALB/c mice are at higher risk of intracellular infections, and tumorigenesis [35–37]. The role of the hematopoietic cell-restricted SHIP as a critical regulator of myeloid cell development and activation has been well established in C57BL/6 mice, but less is known about the role of SHIP in BALB/c mice. Moreover, since SHIP-deficiency on the C57BL/6 background alters T_H_1/T_H_2 balance in these mice (i.e., enhances M2-skewing of Mϕs), the role of SHIP in the already T_H_2-skewed BALB/c microenvironment is particularly intriguing.

As presented herein, we found that deletion of SHIP in BALB/c mice, while not as deleterious as in C57BL/6 mice, leads to a consistent, observable phenotype that is characterized by pulmonary inflammation, myeloid hyperplasia, and M2 skewing of Mϕs (Fig. 1). Moreover, SHIP−/− myeloid cells are significantly more immunosuppressive toward TCR-stimulated T cell proliferation than their WT counterparts (Fig. 1I). These data run counter to the SHIP−/− BALB/c phenotype observed by Maxwell *et al.* [13], and there are other notable differences between SHIP−/− C57BL/6 and SHIP−/− BALB/c colonies from our two groups. For example, Maxwell *et al.* reported that the median survival of SHIP−/− mice on a mixed 129 × C57BL/6 background under specific pathogen-free conditions could be dramatically improved (from 84 to 200 days) if the mice are housed on dust-free bedding [13], but using 3 different commercially available dust-free beddings with our specific pathogen-free colony of SHIP−/− 129 × C57BL/6 mice we did not observe an increase from a median survival time of 84 days (data not shown). As well, they reported that survival improved with backcrossing onto either the C57BL/6 or BALB/c backgrounds [13], but we found survival only improved when backcrossed onto the BALB/c background and actually worsened when backcrossed onto the C57BL/6 background (data not shown). The considerable differences between our SHIP−/− colonies and those of Maxwell *et al.* is an open question, although it is worth noting that our findings are consistent with Roongapinun *et al.* [10], who found that although SHIP−/− BALB/c mice possess mild spontaneous lung inflammation, they exhibit a reduced lung inflammatory response when challenged with ovalbumin, likely due to more immunosuppressive cells. When taken with these observations, our data indicate that the genetic background has a strong influence on the severity and repercussions of deleting SHIP, and also that the loss of SHIP is sufficient to cause an inflammatory lung phenotype even in BALB/c mice.

It was somewhat surprising that SHIP deficiency induced a less severe phenotype in BALB/c mice than C57BL/6 mice. T_H_2 cytokines (i.e., IL-4, IL-10, IL-13, TGF-β, PGE_2_) drive myeloid cell expansion and M2-polarization as part of the physiological wound healing response and thus we originally hypothesized that inherently T_H_2-skewed BALB/c mice would be more sensitive to the development of a myeloproliferative phenotype. However, while SHIP deletion in C57BL/6 mice caused weight loss, frail physique, and decreased lifespan due to massive myeloid hyperplasia and pulmonary infiltration [5–7], SHIP−/− BALB/c mice exhibited relatively mild myeloid hyperplasia and pulmonary lung disease that did not have an obvious effect on mouse health or lifespan (Fig. 1A, C). It is possible that the BALB/c T_H_2 background may actually desensitize mice to the additional M2/T_H_2-promoting effects of SHIP deletion due to compensatory mechanisms and, related to this, BALB/c mice have been shown to possess more Tregs and more suppressible CD4^+^ T cells than C57BL/6 mice [38]. Nevertheless, it is intriguing that the deletion of SHIP in these two disparate genetic backgrounds produced such similar phenotypes, albeit with much different severities. Although the molecular mechanisms by which SHIP restricts M2 polarization in this model are not fully elucidated, we previously found in C57BL/6 mice that SHIP deficiency allows increased PI3K activation in basophils, which results in higher levels of basophil-produced IL-4, and increased M2 polarization of Mϕs [39]. Both SHIP−/− BALB/c and C57BL/6 mice exhibited enhanced myeloid cell expansion, immunosuppressive function, M2-skewing, and lung inflammation, indicating that SHIP restricts myeloid cell development and alternative activation in both genetic backgrounds.

SHIP serves as a tumor suppressor of hematologic malignancies [14] by directly restraining the PI3K pathway in leukemic cells [15–19]. It is worth mentioning that the expansion of myeloid cells, both in myeloproliferative diseases [40] and in response to dexamethasone treatment [41], has been linked to increases in microRNA (miR)-155, which is thought to primarily target SHIP [42]. The potential role of SHIP in solid tumors has not been extensively studied, although the role of SHIP in regulating the development and function of immune modulatory cells that can create an immunosuppressed, pro-tumorigenic environment suggests that loss of SHIP might promote primary tumor growth [21, 22]. SHIP−/− BALB/c mice had not been previously utilized for tumor studies, and therefore both the impact of tumors on the SHIP−/− BALB/c phenotype and the effect of SHIP deletion on tumor growth in BALB/c mice were unknown. We found that SHIP−/− mice orthotopically implanted with non-metastatic 67NR mammary tumors exhibited only a minor change in phenotype; the presence of 67NR tumors did not alter the number of total cells (Fig. 2A) or myeloid cells (Fig. 1F, 2C) in the lungs, Mϕ phenotype (Fig. 1G, 2E), or mouse weight (Fig. 2G), but did cause modest splenomegaly (Fig. 2B) and induced the development of immunosuppressive MDSCs (Fig. 2F). Consistent with this, SHIP−/− or WT mice with 67NR tumors survive beyond 4 weeks of tumor implantation with no apparent pathologies other than a large primary tumor.

Interestingly, we found that metastatic 4T1 tumors (which recapitulate stage IV human breast cancer by metastasizing to vital organs) severely exacerbated the SHIP−/− BALB/c phenotype, causing red and inflamed ears, paws and tails, rapid weight loss (Fig. 3A), and death by 14-17 days after tumor implantation due to massive necrohemorrhagic inflammation of the lungs (Fig. 5A, 5B). It is important to note that this phenotype was not exhibited by WT mice bearing 4T1 tumors (Fig. 3A, 5A, 5B), which were able to survive at least 28 days after primary tumor implantation (when the tumors reached ethical size restrictions). Furthermore, the presence of 4T1 mammary tumors in SHIP−/− mice created a massive expansion of pulmonary myeloid cells relative to naïve (tumor-free) mice or 4T1-bearing WT mice (Fig. 1E, 3D). 4T1 tumors induced a 7-fold increase in total pulmonary cells (Fig. 3C), including 17-fold more myeloid cells and 9-fold more Mϕs, than naïve SHIP−/− mice (Fig. 1F, 3D). Unlike in naïve SHIP−/− mice or SHIP−/− mice with 67NR tumors (Fig. 2D–2F), 4T1 tumor-bearing SHIP−/− mice possessed MDSCs that expressed Arg1 (Fig. 4A) and were over 40-fold more immunosuppressive on a per cell basis than MDSCs isolated from 4T1 tumor-bearing WT mice (Fig. 4C). These data indicate that the enhanced immunosuppressive potency of pulmonary myeloid cells and concomitant necrohemorrhagic lung inflammation is driven by a combination of SHIP deficiency and 4T1 tumor-derived factors. Additional analysis of peripheral blood and bronchoalveolar lavage fluid cytokine levels will help determine whether SHIP deficiency induces an increase in the same factors that are produced by 4T1 tumor cells, or perhaps increases sensitivity to 4T1-produced cytokines.

The specific factors produced by 67NR and 4T1 mammary tumor lines that are responsible for driving the distinct phenotypic changes in tumor-bearing SHIP−/− mice are the topic of ongoing research, although 4T1 tumor cells are known to be an abundant source of G-CSF [26, 27] and mice with 4T1, but not 67NR, tumors exhibit increased serum levels of G-CSF, but not GM-CSF or M-CSF (Bosiljcic, Hamilton, and Bennewith *unpublished data*). G-CSF is known to induce splenic myelopoiesis, splenomegaly, and expansion of immunosuppressive MDSCs in the circulation and peripheral tissues [43, 44], where they are thought to promote the subsequent growth of mammary tumor metastases [27, 45, 46]. Moreover, Mϕs with potent immune suppressive functions also accumulate in the lungs of mice with metastatic mammary tumors and promote metastatic growth [29]. It is interesting to note that 67NR tumors induced accumulation of immunosuppressive Mϕs in the lungs of WT mice (Fig. 1F, 2C), and that these pulmonary Mϕs are further elevated in SHIP−/− mice (Fig. 2C), even though 67NR tumors are unable to disseminate from the primary tumor and, therefore, are not metastatic. Thus, while our previous data indicate that metastatic mammary tumors induce pulmonary accumulation of MDSCs and Mϕs that promote metastatic tumor growth [29], the presence of Mϕs in the lungs alone does not indicate that metastatic tumor cells are present or that the primary tumor itself is metastatic.

Importantly, our findings provide the first evidence that SHIP inhibits the development and growth of pulmonary mammary tumor metastases. While SHIP deficiency did not alter the growth of either 67NR (Fig. 2H) or 4T1 (Fig. 5C) primary tumors, we observed nearly 8-fold more 4T1 tumor cells in the lungs of SHIP−/− mice (Fig. 5E). Tumor-induced myeloid cells play critical roles in the promotion of tumorigenesis via multiple mechanisms, including promotion of angiogenesis, stromal formation and remodeling, and suppression of anti-tumor immunity [24, 25, 46]. The robust expansion of tumor-promoting, immune suppressive myeloid cells in 4T1 tumor-bearing SHIP−/− mice is consistent with the increase in pulmonary metastases we observed in these mice. SHIP restricts the M2 polarization of Mϕs in 4T1 tumor-bearing mice, which is particularly important since M2-Mϕs are key promoters of tumorigenesis in this model and there have been a number of recent reports that restricting alternative activation of Mϕs can decrease 4T1 tumor growth and metastasis [28, 47–49]. As well, it is noteworthy that SHIP deficiency preferentially increases the levels of M-MDSCs and Mϕs (Fig. 3E, Supplemental Fig. 3C), since M-MDSCs are more immunosuppressive than G-MDSCs [50–52] and our previous work shows that Mϕs are up to 30-fold more potent suppressors of activated T cell proliferation than MDSCs in tumor-bearing WT BALB/c mice [29]. It is also intriguing that SHIP restricts mammary tumor metastases but has no effect on primary mammary tumor growth. These data may indicate that myeloid cells play a more critical role in promoting metastatic growth of 4T1 tumors that metastasize to the lungs than the growth of established primary tumors, which is consistent with our previous finding that differentiation of MDSCs into more potently immunosuppressive Mϕs with all-trans retinoic acid enhanced pulmonary metastases without altering the growth of primary 4T1 tumors [29].

Collectively, our data indicate that deletion of SHIP in BALB/c mice induces mild myeloid hyperplasia and inflammatory lung disease, and that this baseline pathology is dramatically amplified during the growth of 4T1 metastatic mammary carcinoma. We found that SHIP restricts myeloid cell expansion, alternative activation, and immunosuppressive function in both naïve and tumor-bearing BALB/c mice, and represses the metastasis of mammary tumors. These findings support the concept that pulmonary inflammation helps to create a pro-metastatic environment in the lungs, and suggest that small molecule activators of SHIP may be a therapeutically useful strategy to restrict the accumulation and activation of tumor-induced myeloid cells in tissues and aid in the prevention or treatment of metastases in breast cancer patients.

## MATERIALS AND METHODS

### Mice and tumor models

F2 SHIP−/− mice on a mixed C57BL/6 × 129Sv background were backcrossed onto a BALB/c or C57BL/6 background for 10 generations. Mice heterozygous for SHIP were bred and F10 SHIP WT and SHIP−/− female mice between 8-14 weeks of age were used for all experiments. 67NR and 4T1 murine mammary carcinoma cells (kind gifts from Dr. Fred Miller, Karmanos Cancer Institutes, Detroit, MI) were maintained in RPMI-1640 medium + 10% FCS and used within 20 passages. These cell lines were derived from a spontaneous mammary tumor in a BALB/cfC3H mouse and represent different levels of metastatic propensity; 67NR cells do not metastasize, while 4T1 tumor cells metastasize to the lung, liver, bone, and brain [53]. Mice were orthotopically injected with 2×10^5^ 67NR or 10^5^ 4T1 cells in the fourth mammary fat pad (cell numbers were determined to produce tumors with similar growth rates that approach ethical restrictions 3-3.5wks after implantation). 67NR and 4T1 tumor-bearing mice were sacrificed 21 days or 15-17 days after tumor implantation, respectively. Mice were housed under specific-pathogen free conditions in the Animal Resource Centre at the BC Cancer Agency Research Centre. All animal experiments were performed in accordance with Institutional and Canadian Council on Animal Care Guidelines.

### Myeloid cell isolation

To prepare single-cell suspensions, spleens were passed through a 70 μm filter, while lungs were finely minced prior to agitation for 40 min at 37°C with 0.5% trypsin (BD Biosciences, Mississauga, ON, Canada) and 0.08% collagenase in PBS. After incubation, 0.06% DNase was added, and the cell suspensions filtered through 30 μm nylon mesh. Unless otherwise stated, all tissue culture reagents were from StemCell Technologies (Vancouver, BC, Canada) and all other reagents from Sigma-Aldrich (St. Louis, MO).

Both Gr1^+^ cells and F4/80^+^ cells were isolated from single cell suspensions using PE positive selection EasySep magnetic bead-assisted isolation systems (StemCell Technologies), according to the manufacturer's instructions. Gr1^+^ cells were found to be >95% CD11b^+^Gr1^+^ and F4/80^+^ cells were >95% CD11b^+^F4/80^+^ by flow cytometry. Mϕs were obtained by lavage of the peritoneal cavity with 3 × 5 ml HL-1 medium (BioWhittaker, Basel, Switzerland) + 1 mM EDTA. Peritoneal Mϕs were resuspended in HL-1 medium without EDTA, plated, and allowed to adhere for at least 3 h at 37°C before non-adherent cells were washed away. Analysis of the adherent cells revealed that >95% were Mϕs, co-expressing F4/80 and CD11b and exhibiting characteristic Mϕ morphology. To assess morphology, cytospin preparations of Gr1^+^ lung cells were stained with Giemsa-Eosin. Images were captured with a Retiga EXi camera (QImaging, Surrey, BC, Canada) using an Axiovert S100 microscope (Carl Zeiss Canada Ltd., Toronto, ON, Canada).

### Flow cytometry

Cells were suspended in Hank's Balanced Salt Solution + 2% FCS + 0.05% NaN_3_ and blocked with 1 μg rat anti-mouse CD16/CD32 Ab (2.4G2) (BD Biosciences) for 10 min at 4°C. Cells were incubated for 30 min at 4°C with APC-, FITC-, or PE- conjugated antibodies specific for mouse CD11b (eBioscience, San Diego, CA), Gr1 (eBioscience), F4/80 (Invitrogen, Burlington, ON, Canada), B220 (BD Biosciences), CD3 (BD Biosciences), Ly6C (BD Biosciences), or Ly6G (BD Biosciences). Data were acquired using a FACSCalibur flow cytometer (BD Biosciences) and analyzed using FlowJo software (Tree Star, Inc., Ashland, OR). Absolute numbers of cells were calculated by multiplying the proportion of a particular cell type as determined by flow cytometry by the total number of cells recovered from disaggregated tissue.

### Western blots

Cells were washed with PBS and lysed with 1xSDS sample buffer. Samples were boiled for 2 min and loaded onto 10% polyacrylamide gels and subjected to SDS-PAGE and Western blot analysis as described previously [54]. Antibodies against SHIP (P1C1; Santa Cruz Biotechnology, Santa Cruz, CA), Arg1 (BD Biosciences), and GAPDH (Fitzgerald Industries International, Acton, MA) were used.

### Cytokine assays

Peritoneal Mϕs were stimulated with 100 ng/mL LPS for 3 h and cell-free supernatants collected. IL-10 and IL-12p40 production was assayed using cytokine ELISA kits (BD Biosciences), according to the manufacturer's instructions.

### T cell proliferation assays

Assays were performed using HL-1 serum-free medium supplemented with 1% penicillin, 1% streptomycin, 1% Glutamax, and 5 × 10^−5^ M 2-ME [54]. Erythrocyte-depleted WT syngeneic splenocytes were cultured at 2×10^5^ cells/well ± myeloid cells and stimulated with 1 μg/ml anti-CD3 + 5 μg/ml anti-CD28 (eBioscience). Cells were incubated at 37°C for 72 h and 1 μCi/well ^3^H-thymidine (2 Ci/mM; PerkinElmer, Woodbridge, ON, Canada) was added for the last 18 h. Cells were harvested onto filtermats and radioactivity measured using a Betaplate liquid scintillation counter (Wallac, Waltham, MA). Assays were performed in triplicate and data expressed as the fraction of stimulated T cell proliferation in the absence of myeloid cells.

### Histology

Lungs and spleens were formalin fixed, ethanol washed, and paraffin embedded. Sections were obtained and stained with H&E, using standard techniques. Slides were scanned at 20X using a Hamamatsu Nanozoomer scanner and images obtained from the scanned slides at magnifications indicated by the scale bars. All slides were interpreted by a Board Certified Veterinary Pathologist (Dr. Meegan Larsen, MBed Pathology).

### Clonogenic assays

Monodispersed lung cells (derived by enzymatic disaggregation of lung tissue as outlined above) were washed by centrifugation prior to NH_4_Cl erythrocyte lysis. Cells were washed in PBS, resuspended in medium, and aliquots of 3×10^3^ to 1×10^6^ cells plated in clonogenic assays containing 250 μg/ml geneticin or 60 μM 6-thioguanine (to specifically allow growth of 67NR and 4T1 cells, respectively). Cells were incubated for 9-12 days (37°C, 5% CO_2_) prior to staining colonies with malachite green for enumeration. The total number of clonogenic tumor cells in the lungs was calculated by multiplying the proportion of colony forming tumor cells by the total number of cells recovered from the lungs.

### Statistics

Unless otherwise stated, data are expressed as the mean ± SEM of triplicate determinations and are representative of two or three independent experiments. Student t tests were performed using Microsoft Office Excel 2007. Differences p <0.05 were considered significant; */#,p <0.05; **/##,p <0.01; ***/###,p <0.001.

## SUPPLEMENTARY FIGURES


