# Supplementary material for: SHIP represses lung inflammation and inhibits mammary tumor metastasis in BALB/c mice
Source: Oncotarget. 2015 Dec 14;7(4):3677–91. doi: 10.18632/oncotarget.6611 (PMC4826161; doi:10.18632/oncotarget.6611)
Supplement: Supplementary file 1 [file oncotarget-07-3677-s001.pdf]

SUPPLEMENTARY FIGURES

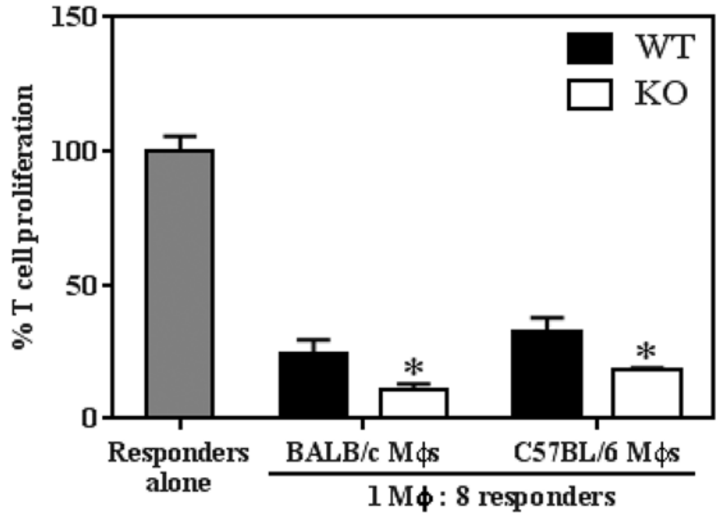

Supplementary Figure S1: SHIP restricts the immunosuppressive potencies of both BALB/c and C57BL/6 Mϕs. Peritoneal Mϕs were isolated from WT and SHIP-/- BALB/c and C57BL/6 mice and co-cultured with anti-CD3 + anti-CD28 stimulated splenocytes (1 Mϕ:8 splenocytes). Data are expressed as the fraction of stimulated T cell proliferation in the absence of Mϕs (responders alone). *$p<0.05$ compared to WT.

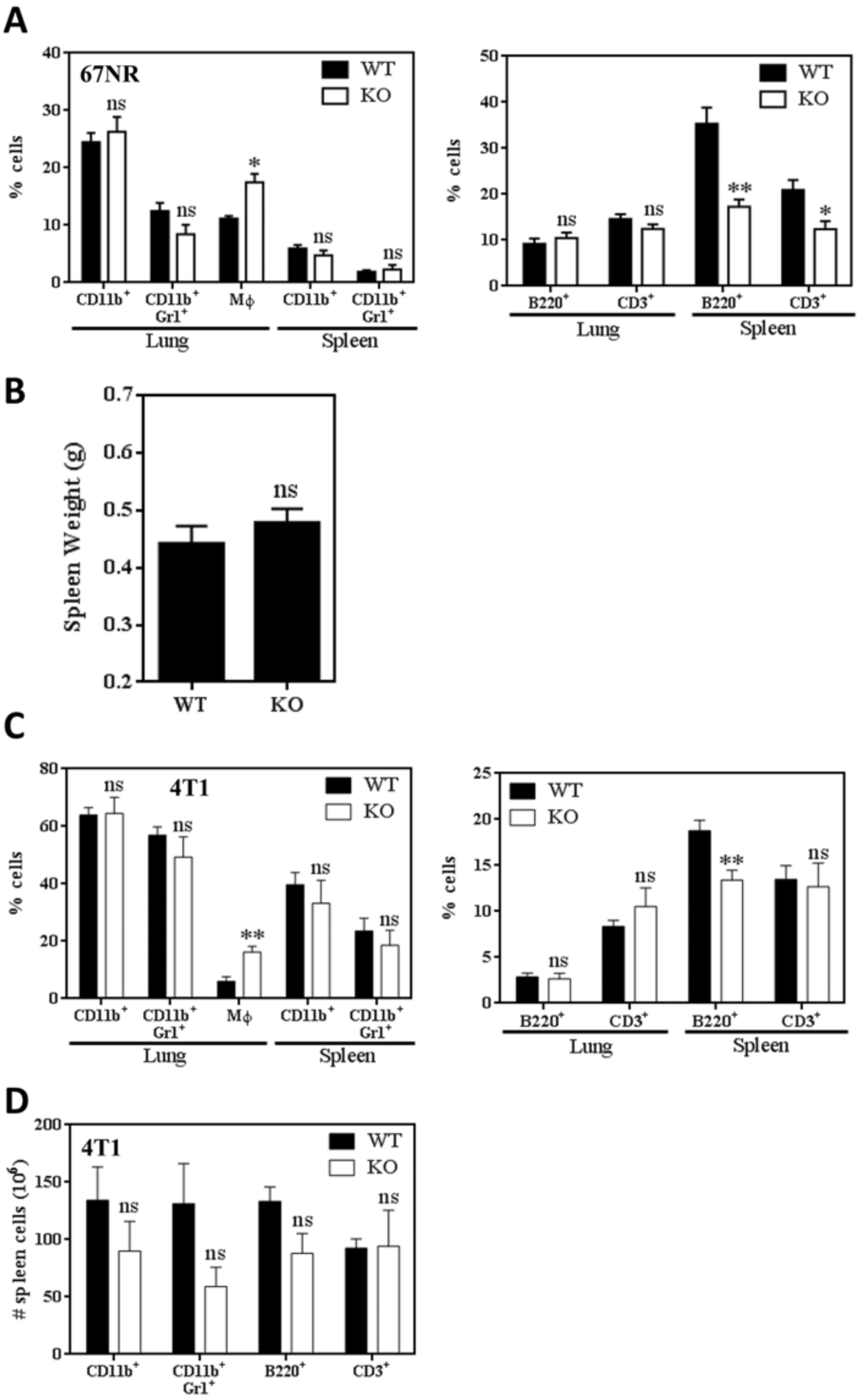

Supplementary Figure S2: SHIP deficiency alters the proportions of myeloid and lymphoid cell types in naïve, 67NR, and 4T1 tumor-bearing BALB/c mice. A. Proportion of CD11b$^+$ cells, CD11b$^+$Gr1$^+$ myeloid cells, and CD11b$^+$F4/80$^+$ Mϕs (*left*) or B220$^+$ B lymphocytes and CD3$^+$ T lymphocytes (*right*) in the lungs and spleens of WT and SHIP-/- 67NR tumor-bearing mice. Data are expressed as mean ± SEM with 3-4 mice per group. **B.** Spleen weights of WT and SHIP-/- BALB/c mice 15 days after orthotopic 4T1 tumor implantation. **C.** Proportion of CD11b$^+$ cells, CD11b$^+$Gr1$^+$ myeloid cells, and CD11b$^+$F4/80$^+$ Mϕs (*left*) or B220$^+$ B lymphocytes and CD3$^+$ T lymphocytes (*right*) in the lungs and spleens of WT and SHIP-/- 4T1 tumor-bearing mice. **D.** Numbers of splenic myeloid cells and lymphocytes in WT and SHIP-/- 4T1 tumor-bearing mice. A-D, Data are mean ± SEM of two independent experiments with 8-10 mice per group. *p<0.05; **p<0.01; ns, no significant difference. Significance compared to WT.

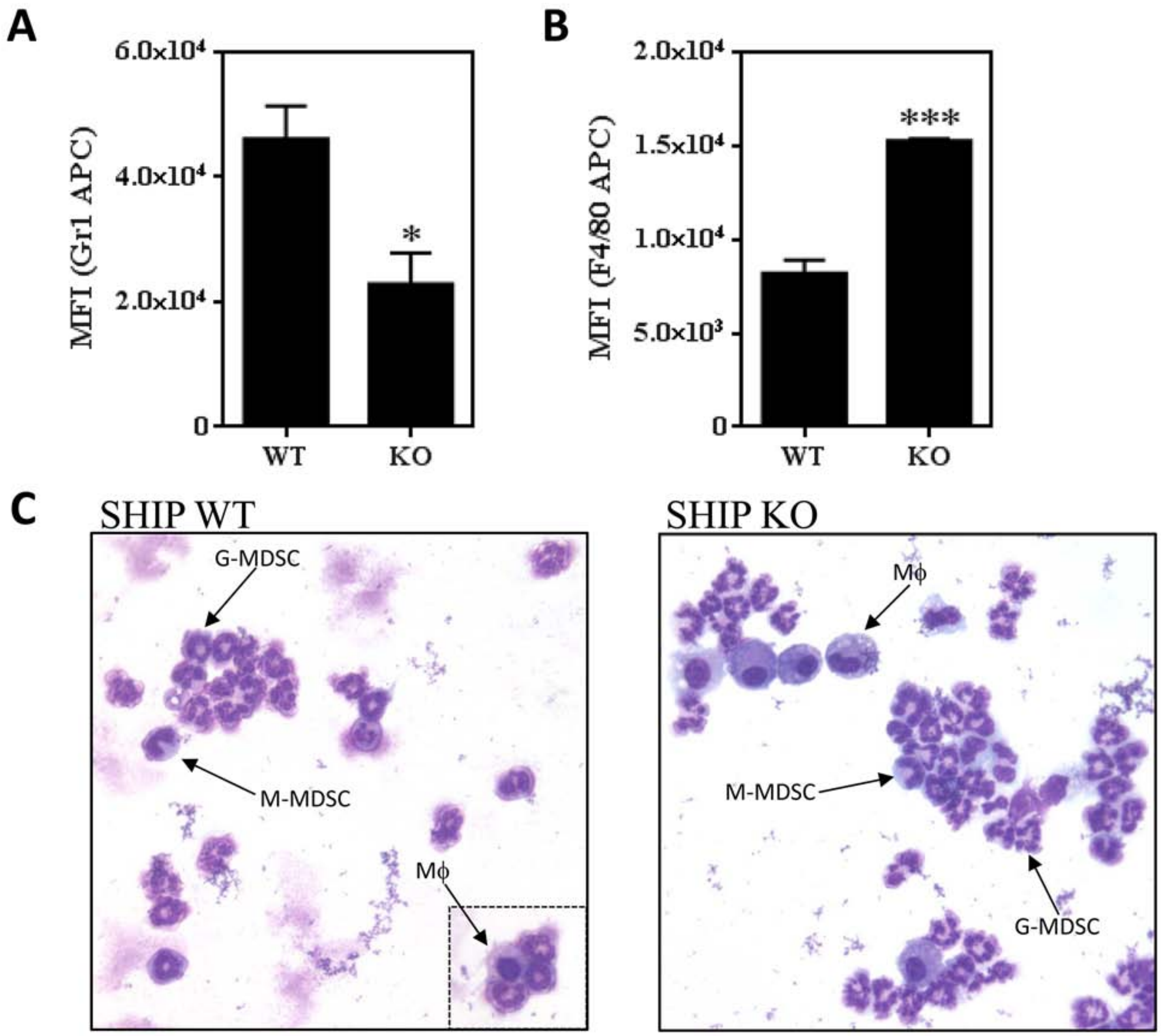

Supplementary Figure S3: 4T1-induced myeloid cells from WT and SHIP-/- BALB/c mice differentially express Gr1 and F4/80. A. Mean fluorescence intensity of Gr1 expression by $CD11b^{+}Gr1^{+}$ cells isolated from the lungs of WT and SHIP-/- 4T1 tumor-bearing mice. **B.** F4/80 expression by $CD11b^{+}F4/80^{+}$ cells isolated from the lungs of WT and SHIP-/- 4T1 tumor-bearing mice. A-B, Data are expressed as mean ± SEM with 3 mice per group. **C.** Morphological analysis of $Gr1^{+}$ pulmonary cells isolated in (A) reveals three distinct subpopulations: granulocytic cells (G-MDSCs), monocytic cells (M-MDSCs), and Mϕs. Inset WT image (*dashed line*) is from a separate field of view on the same slide. Images are of Giemsa-stained cytospins viewed with a 40X objective and are representative of two independent experiments, each with 3 mice per group.

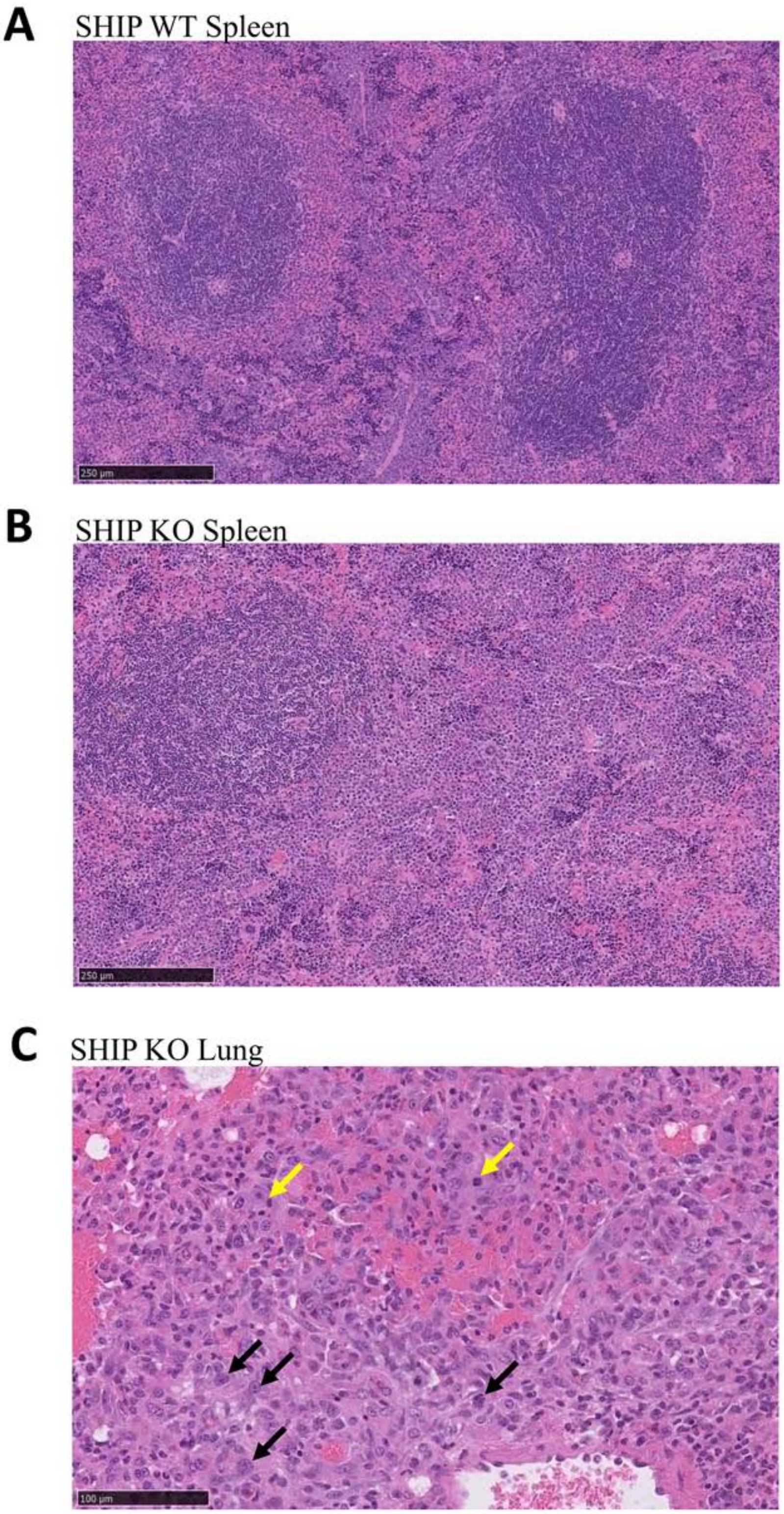

Supplementary Figure S4: 4T1 tumor-bearing SHIP-/- BALB/c mice exhibit lung metastases and severe splenic myeloid hyperplasia. A. Spleens of 4T1 tumor-bearing WT mice contain defined regions of white and red pulp and moderately increased numbers of both myeloid and erythroid precursors. **B.** Spleens of 4T1 tumor-bearing SHIP-/- mice contain expanded regions of red pulp and markedly increased levels of myeloid cells, while the lymphoid-containing white pulp is depleted. **C.** Lungs of 4T1 tumor-bearing SHIP-/- mice contain metastatic mammary tumor cells (*black arrows*), a few of which exhibit mitotic figures (*yellow arrows*). Images are representative of 2 mice from each group 15 days after orthotopic 4T1 tumor implantation; scale bars are 250µm in **A** and **B**, and 100µm in **C**.
